# Sea Ice Detection Based on an Improved Similarity Measurement Method Using Hyperspectral Data

**DOI:** 10.3390/s17051124

**Published:** 2017-05-15

**Authors:** Yanling Han, Jue Li, Yun Zhang, Zhonghua Hong, Jing Wang

**Affiliations:** College of Information Technology, Shanghai Ocean University; Shanghai 201306, China; ylhan@shou.edu.cn (Y.H.); lijue93@163.com (J.L.); zhhong@shou.edu.cn (Z.H.); wangjing@shou.edu.cn (J.W.)

**Keywords:** sea ice, similarity measure, band selection, classification, hyperspectral image

## Abstract

Hyperspectral remote sensing technology can acquire nearly continuous spectrum information and rich sea ice image information, thus providing an important means of sea ice detection. However, the correlation and redundancy among hyperspectral bands reduce the accuracy of traditional sea ice detection methods. Based on the spectral characteristics of sea ice, this study presents an improved similarity measurement method based on linear prediction (ISMLP) to detect sea ice. First, the first original band with a large amount of information is determined based on mutual information theory. Subsequently, a second original band with the least similarity is chosen by the spectral correlation measuring method. Finally, subsequent bands are selected through the linear prediction method, and a support vector machine classifier model is applied to classify sea ice. In experiments performed on images of Baffin Bay and Bohai Bay, comparative analyses were conducted to compare the proposed method and traditional sea ice detection methods. Our proposed ISMLP method achieved the highest classification accuracies (91.18% and 94.22%) in both experiments. From these results the ISMLP method exhibits better performance overall than other methods and can be effectively applied to hyperspectral sea ice detection.

## 1. Introduction

Sea ice is the cause of most marine disasters in polar and high latitude regions. As a component of the global marine and atmospheric system, sea ice with high albedo impacts marine industries, and matter and momentum exchanges between the atmosphere and the ocean. In addition, sea ice plays a key role in the balance of radiation, energy, and mass on ocean surfaces [1,2]. Therefore, studying changes in sea ice is highly important. However, because of the harsh natural environment in areas covered with sea ice, conventional observation methods such as the in situ sampling method and the visual estimate method [3] cannot acquire detailed information on sea ice changes in a timely and effective manner. Instead, remote sensing technology, which can analyze data for large areas of sea ice rapidly and extensively, is widely used in sea ice detection. The data sources commonly used include the National Oceanic and Atmospheric Administration (NOAA)/advanced very-high-resolution radiometer (AVHRR) [4], and AQUA and TERRA satellite/moderate-resolution imaging spectroradiometer (MODIS) [5] images, etc. Due to the limitations of spectral resolution, these sea ice detection methods primarily focus on the extraction of sea ice extent. It is difficult to acquire further details about the sea ice, such as class. Compared with the multispectral technologies, hyperspectral remote sensing technology can acquire nearly continuous spectrum information and rich sea ice image information, providing an important resource for sea ice detection. Widely used sea ice detection methods include the threshold method, unsupervised classification and supervised classification [6,7] such as in the K-means and support vector machine (SVM) methods. 

A hyperspectral data set contains hundreds of spectral bands characterized by large amounts of data and narrow bands (high spectrum bandwidth is usually less than 10 nm) [8]. Different types of sea ice in band ranges such as those from 400 to 610 nm exhibit remarkable reflectivity differences and strong separability [9]. Applied to sea ice detection, the abundant spatial and spectral information of the hyperspectral data offers huge potential; however, the accompanying surge in data volume introduces large challenges to data processing and interpretation methods. Also, the high correlation and redundant information in the bands can significantly reduce the precision achievable by the traditional image classification methods [10]. A data dimensionality reduction can reduce the amount of processing required on the original data under the premise that it reserves the important information. As a result, the dimensionality reduction of hyperspectral sea ice data is of great significance for subsequent processing.

The existing dimensionality reduction methods are primarily divided into two categories [11]: methods based on feature extraction, such as principal component analysis (PCA) [12], and methods based on band selection, such as entropy [13]. The methods based on feature extraction change the physical meaning of the original band during the feature-extraction process; consequently, they cause the loss of information to a certain extent. In contrast, the band selection-based methods retain the original band characteristics without affecting their physical meaning; thus, they are widely used in hyperspectral data dimensionality reduction. Numerous band selection methods have been proposed recently. Based on whether they require prior knowledge, these band selection methods can be divided into two major types: supervised and unsupervised. Unsupervised band selection methods do not require prior knowledge about ground objects; therefore, this method is widely applied to remote sensing image processing.

The basic idea of unsupervised band selection methods is to calculate the amount of information and the similarity between different bands to identify the most distinctive and informative bands [14]. Of the methods based on information sorting, such as entropy and adaptive band selection (ABS), entropy was used to detect redundancy between bands in [13], while ABS was investigated in [15]. Of the methods based on minimum error band refactoring such as linear prediction (LP) and first spectral derivative (FSD) [16], two modified LP band selection methods were proposed for unsupervised band selection in [17]. However, these methods have some shortcomings. For instance, the method based on entropy takes into consideration only the amount of information in the band itself, ignoring the correlation between bands, resulting in a non-optimal subset of selected bands. In contrast, the LP-based methods primarily consider the similarity between bands without fully considering the information in the bands themselves. To a certain extent, this approach affects the final classification accuracy.

To choose the optimal band combination that features both a large amount of information and a low-degree of inter-band correlation, this paper proposes an improved similarity measurement method based on LP (ISMLP) for hyperspectral sea ice detection. Firstly, we chose the first band with maximum amount of information based on mutual information (*MI*) theory [18], and then determined the second band with the least similarity by the spectral correlation measure (SCM) method [19], finally, we chose the subsequent band through the LP method, and used a support vector machine model for sea ice classification. The experimental analysis is carried out on Baffin Bay data and Bohai Bay data.

The paper is organized into four sections. In the next section, the improved similarity measurement method is proposed, the general framework of sea ice detection based on ISMLP method is presented, and the major issues about method implementation are investigated, such as pixel selection, band selection method and the sea ice classification model. Section 3 illustrates the experimental analysis. Finally, Section 4 presents the main contributions and draws the conclusions of this paper.

## 2. Improved Similarity Measurement Method 

The purpose of band selection is to choose the optimal band combination from the available bands that contain a large amount of information but low band similarity. In this paper, band selection was achieved by using LP to perform a similarity comparison. Because LP is sensitive to the original bands, choosing the original bands with a large amount of information and low similarity is crucial. As the first step, the *MI* theory, which performs well in terms of information measurement, was used to determine the first original band containing a large amount of information. High-spatial-resolution Landsat images were used as a benchmark. Then, in the second step, the SCM method was utilized to obtain the second original band because the method has demonstrated good performance in terms of spectrum recognition. Finally, subsequent bands were obtained using the LP method. Then, the SVM classifier model [20] was applied to classify the sea ice using the selected bands. Data preprocessing was required during the method implementation to determine the band range with good separability for sea ice, to remove poor bands and so on. In addition, we also analyzed the influence of different proportions of pixel selection on the process of band selection, and studied the number of selected bands required after dimensionality reduction. We compared this approach with traditional band selection methods such as entropy, LP, and ABS. The results of these experiments indicate that, overall, the proposed method achieves a better classification performance than do other traditional methods, which indicates that this method can be effectively applied in hyperspectral sea ice detection.

**Figure 1:**
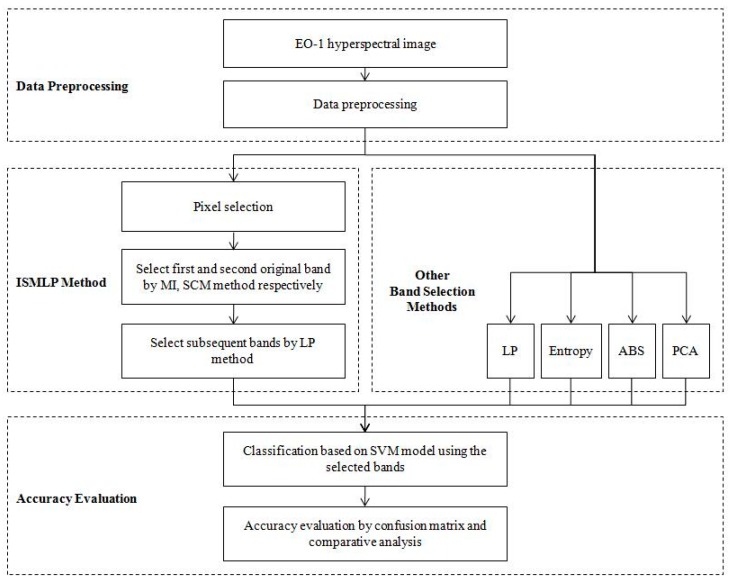
General framework for hyperspectral sea ice detection based on the improved similarity measurement method based on linear prediction (ISMLP) method. LP: linear prediction; SCM: spectral correlation measure; MI: mutual information; ABS: adaptive band selection; PCA: principal component analysis; SVM: support vector machine; EO-1: Earth-Observing-1.

Figure 1 shows the entire framework for detecting sea ice based on the proposed ISMLP method. Four major issues were investigated: (1) original band selection, (2) subsequent band selection, (3) hyperspectral sea ice image classification, and (4) classification accuracy evaluation. The methods are discussed in detail in the following sections.

### 2.1. Pixel Selection

Due to the large number of pixels in a remote sensing image, the sizes of the matrices in the data process are large, which reduces efficiency. However, using only a relatively small subset of pixels in the band selection process does not change the results in most cases [14]. This is because a high spatial correlation exists among bands of hyperspectral data. Therefore, this study made a comparative analysis of different pixel-selection proportions.

- Number of selected pixels: First select a band with all pixels, followed by a comparative analysis using all pixels, 1/100 pixels, 1/1000 pixels, 1/10,000 pixels, etc.
- Locations of the selected pixels: When using a low proportion to randomly select pixels for an entire image, not all categories of pixels can always be included in all cases. To eliminate this effect, this investigation adopted a pixel-selection method using K-means clustering. The specific steps are as follows:
  1 Select all the bands (after removing the invalid bands without radiometric calibration), perform the K-means clustering classification, and then merge the same categories.
  2 Calculate the number and locations of different categories of pixels, and determine the number of pixels in each category.
  3 According to step 2, choose the corresponding pixels in each type by uniform randomness.

In the experiments, the ISMLP method was employed to perform the comparative analysis using different pixel-selection proportions. Through experimental analysis, this study found that the pixel-selection method based on K-means clustering [21] selects corresponding pixels based on their proportions in different categories. This significantly improves the computational efficiency; consequently, it can achieve similar or higher classification accuracies than using the original data.

### 2.2. Band Selection Method

#### 2.2.1. Original Band Selection Method

The Landsat data, which has high spatial resolution at the same times and the same locations, was adopted as the base band; then, the band with the highest similarity to the base band was chosen by *MI* as the first initial band. *MI* is a basic concept of information theory. It describes the statistical correlation between two random variables, that is, a variable exists in the information of another variable [22]. Assuming that the higher resolution benchmark image is taken as the base band *B*_0_, the *MI* between any band *A* and *B*_0_ can be expressed as follows:

(1) $$MI(A,\;B_{0}\;)=\sum p(A,\;B_{0})\cdot \log\frac{p(A,\;B_{0})}{p(A)\cdot p(B_{0})}$$

where *p*(*A*) and *p*(*B*_0_) are the respective probability distributions of *A* and *B*_0_, and *p*(*A*, *B*_0_) is the joint probability distribution between them. *MI* can be used as a similarity measure criterion: the greater the *MI* is, the more information from one band exists in another band. The similarity between the two bands will be higher too. High similarity to the benchmark band indicates that the band contains a greater amount of information. Therefore, the band with the maximum *MI* value can be selected as the first original band.

To choose the second original band that is the least similar to the first original band, the *SCM* method is used to calculate the cross-correlation between two bands. The *SCM* method can be described as follows:

Assuming that *B*_1_ and *B*_2_ are two bands from the set *Φ*, $B_{1}\;=\;(b_{11},b_{12},\ldots ,b_{1L})$ and $B_{2}\;=\;(b_{21},b_{22},\ldots ,b_{2L})$, and *L* is the number of pixels, then the cross-correlation between two bands is:

(2) $$SCM(B_{1},B_{2})=\frac{\sum_{i=1}^{L}\left(b_{1i}-\bar{B_{1}}\right)(b_{2i}-\bar{B_{2}})}{\sqrt{{\sum_{i=1}^{L}\left(b_{1i}-\bar{B_{1}}\right)}^{2}}\sqrt{{\sum_{i=1}^{L}\left(b_{2i}-\bar{B_{2}}\right)}^{2}}}$$

where $\bar{B_{1}}$ and $\bar{B_{2}}$ are the averages of bands *B*_1_ and *B*_2_, respectively. The greater the value is, the higher the similarity between the two bands is. Then, we choose the least-similar band as the second original band.

This method takes both the band information and the similarity between the bands into consideration when choosing the initial band pair. Thus, it chooses a more optimized initial band pair, and provides a better method for subsequent band selection.

#### 2.2.2. Subsequent Band Selection Method

The basic steps of the subsequent band selection method are as follows [23]:

- Initialize the algorithm with the pair *B*_1_ and *B*_2_, and construct a band subset *Φ* = {*B*_1_, *B*_2_}.
- Find *B*_3_, which is the band least similar to *B*_1_ and *B*_2_, and then update the selected band subset to $\Phi =\Phi \cup \left\{B_{3}\right\}$.
- Repeat until the subset *Φ* contains a sufficient number of bands.

In subsequent band selection, LP [16] is used to calculate the similarity between a single band and multi-bands. LP is defined as follows:

Assuming that bands *B*_1_ and *B*_2_ are the two bands in the subset *Φ*, *B*_1_ and *B*_2_ can be used to calculate band *B*, which is the least similar to bands *B*_1_ and *B*_2_:

(3) $$a_{0}+a_{1}B_{1}+a_{2}B_{2}=B\prime$$

In Equation (3), *B*′ is the result of LP estimation of *B* using *B*_1_ and *B*_2_, while *a*_0_, *a*_1_, and *a*_2_ are the parameters of the minimized LP error; the error is $e_{\min}=\left\|B-B\prime \right\|$, and the parameter vector $a={\left(a_{0},a_{1},a_{2}\right)}^{T}$ can be determined by the least squares solution:

(4) $$a={\left(X^{T}X\right)}^{-1}X^{T}y$$

In Equation (4), *X* is the matrix *L* × 3, the first column is 1, the second column contains all the chosen pixels of band *B*_1_, the third column contains all the pixels of band *B*_2_, and *y* is the vector *L* × 1 that includes all the pixels in band *B*. When we find the band with the maximum error, it is considered as the band most dissimilar to *B*_1_ and *B*_2_. Therefore, it can be used as *B*_3_ and added into the subset *Φ*. The method is carried out repeatedly until the number of selected bands in the subset *Φ* meets the requirements for band selection.

#### 2.2.3. Number of Selected Bands

In practical applications, it is difficult to determine the required number of bands. Generally, when an image scene is complicated, such as one that contains numerous categories, it is necessary to choose more bands because the data dimensions must be sufficiently high to accommodate the categories used in detection and classification. The minimum number of hyperspectral bands can be estimated using the virtual dimension (VD) [24] method.

Under normal circumstances, the noise subspace projection (NSP) obtains the largest estimate in the VD method. Therefore, this method is analyzed in this paper. The NSP estimates the noise matrix. Many methods exist for noise covariance matrix estimation, such as the residual analysis method developed by Roger [25] which is particularly suited for use with hyperspectral image data. Therefore, we apply the estimation method based on the residual errors.

### 2.3. Sea Ice Classification Model

The traditional threshold segmentation method is not efficient for addressing high-dimensional data, and it is difficult to obtain the optimal threshold [26]. Because SVM shows outstanding performance in solving small-sample, high-dimension classification problems, we selected SVM as the benchmark classifier to classify sea ice [20].

Assuming that a training sample set *T* consists of *N* independent samples, denoted as ${\left(x_{i},y_{i}\right)}_{i=1}^{N}$, *x_i_* represents the training samples and *y_i_* ∈ {−1, +1} represents the associated labels. The basic idea of SVM is to map the data of the original sample space into a high-dimension feature space introducing a kernel function to find an optimal classification hyperplane that maximizes the margin between the two classes [20].

The classification problem can be transformed into a typical convex programming problem based on the Kuhn–Tucker theorem. Similarly, the convex programming problem can be transformed into the following dual programming problem through the Lagrange multipliers $\alpha_{i}$, which are associated with the original training patterns *x_i_*:

(5) $$\begin{array}{l} J(a)=\sum_{i=1}^{N}\alpha_{i}-\frac{1}{2}\sum_{i=1,j=1}^{N}\alpha_{i}\alpha_{j}y_{i}y_{j}K(x_{i},x_{j})\\ s.t.\;\sum_{i=1}^{N}\alpha_{i}y_{i}=0,\;\alpha_{i}\geq 0;\;i=1,\ldots ,N \end{array}$$

The dual program problem has an optimal global solution. The Lagrange multipliers $\alpha_{i}$ corresponding to the non-support vectors are zero; therefore, the optimal classification decision function for binary classification can be obtained by solving the above problems:

(6) $$f(x)=\mathrm{sgn}(\sum_{SV}\alpha_{i}y_{i}K(x_{i},x)+b)$$

where *SV* is the support vectors set, $\alpha_{i}$ and *b* are the parameters used to determine the optimal classification hyperplane, and *K*(∙,∙) is the kernel function. We chose the radial basis kernel function, which yields a better classification result [20]. The optimal partition hyperplane problem can be settled by solving a dual optimization problem, according to the convex theory. The obtained solution is an optimal global solution.

The SVM is simply one kind of binary classification classifier. Multiclass classification problems rely on transforming binary classification into multiple binary classification problems to be solved. In view of hyperspectral sea ice data characteristics such as high dimensionality, multiple features, and large amounts of data, we use the one-against-one (OAO) method [20] to solve the multiclass problem. In the OAO method, given *k* classes in the data, we construct a classifier between every two categories (e.g., *i* and *j*). Consequently, we must construct k(k−1)/2 binary classifiers altogether, and each binary classifier will determine whether the samples belong to category *i* or category *j*. Corresponding to each decision function of the binary classification problem, the final decision in the OAO strategy is made on a “winner-takes-all” based by building the following maximization:

(7) $$M(x)=\arg\max\_i(\sum_{SV}\alpha_{i}y_{i}K(x_{i},x)+b)$$

where arg is an indicator function, and argmax () is a subscript value that maximizes the value in the parentheses. Namely, the indicator *i* is chosen as the *M*(*x*) value, and *i* is also the subscript of *f*(*x*), which obtains the maximum value in the *k*-decision function. The *i* category is the corresponding type to which the sample points *x* should belong.

## 3. Experimental Analysis

The Earth-Observing 1 (EO-1) spacecraft that was launched by National Aeronautics and Space Administration (NASA) in November 2000 [27] includes a mounted Hyperion sensor. The resulting hyperspectral images have a total of 242 bands; their spatial resolution is 30 m, and their spectrum coverage ranges from 356 nm to 2578 nm. Two hyperspectral images were used in the experiments described in this paper.

### 3.1. Experiment in Baffin Bay

#### 3.1.1. Data Description

The data for the first experiment was acquired on 12 April 2014, from the EO-1 hyperspectral sea ice data without cloud coverage of Baffin Bay, Greenland. The image covers an area whose upper left-hand corner is located at 79°51′27′′ W, 74°16′16′′ N, and whose lower right-hand corner is located at 79°29′20′′ W, 73°57′5′′ N. The data set belongs to the level L1G, a level at which geometric correction, projection registration, and topographic correction have already been made. When no real surface information (ground truth) is available for comparison, a remote sensing image with higher spatial resolution from the same time and location can be regarded as the reference image, and its classification result can be regarded as the real distribution situation of the ground truth [14]. In this experiment, we used Landsat-8 data whose spatial resolution is 15 m from the same time and covering the same location as the validation data.

#### 3.1.2. Preprocessing of Hyperspectral Data

Because the hyperspectral bands from 1 to 7, from 58 to 78, and from 224 to 242 are invalid without radiometric calibration [27], they were not considered for band selection. In addition, a total of 20 bands of EO-1 Hyperion hyperspectral L1G product data were affected by water vapor, namely, from 121 to 127, from 167 to 178, and 224. Furthermore, there were overlapping bands from 56 to 57 and from 77 to 78. These were removed in advance. The remaining 176 bands range from 8 to 57, from 79 to 120, from 128 to 166, and from 179 to 223. From hyperspectral sea ice spectral characteristics [9], we determined that the valid range of wavelengths is from 400 to 1350 nm. The bands in this range include the 92 bands from 8 to 57 and from 79 to 120; therefore, these were taken as the band sources for band selection.

#### 3.1.3. Pixel Selection and Band Selection Based on the ISMLP Method

Because of the very high spatial correlation between these images, to reduce the amount of calculation, only a subset of the pixels needed to be selected for analysis. In the experiment, a comparative analysis was made of band selection with different pixel-selection proportions based on the K-means method, as shown in Table 1.

**Figure 2:**
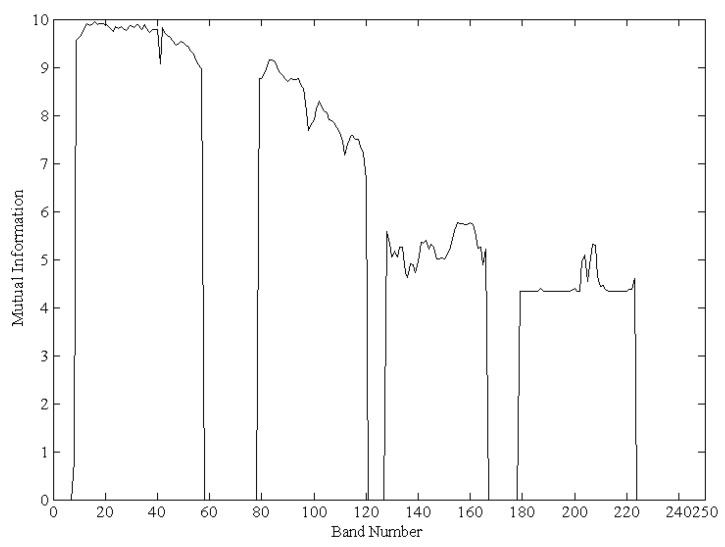
The mutual information (*MI*) of 242 bands between the hyperspectral sea ice image (using all pixels) and the base band.

To choose bands with larger amounts of information and distinctive features, the selection of the initial original band is important because it affects the selection of the subsequent bands and, therefore, has a major effect on the classification results. In this experiment, the higher spatial resolution Landsat-8 data (the spatial resolution is 15 m) taken at the same time and location as the EO-1 images was used as the base band. Then, the *MI* values between every band (using all pixels) and the base band were calculated as shown as in Figure 2.

Figure 2 shows the *MI* value between every band and the base band. The *MI* value is in accord with the characteristics of EO-1 Hyperion hyperspectral L1G product data. It can be seen that the *MI* values of the bands without radiometric calibration are 0. The *MI* value of band 16 has the maximum value of 10.59. Therefore, band 16 was adopted as the initial original band for the ISMLP method. Then, the band that is the least similar was chosen as the second band based on SCM. Finally, the subsequent bands are chosen based on LP. The band selection results by ISMLP for different pixel-selection proportions are shown in Table 1.

**Figure 3:**
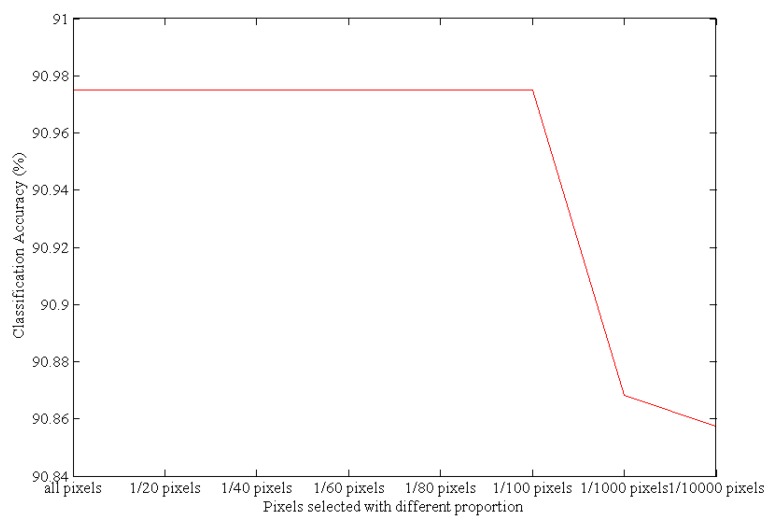
The classification accuracies resulting from different pixel-selection proportions.

As shown in Table 1, the bands selected using the pixel select proportions from all pixels to 1/100 (based on the K-means method) are the same; therefore, the number of pixels can be reduced to one percent. This result indicates that the K-means method is an effective way to select pixels. When using only 1/1000 pixels, the second original band changed and the subsequent bands were not the same as those selected when using all pixels. When using only 1/10,000 pixels, the selected bands changed significantly. This analysis also serves to show that using a relatively small subset of pixels during the band selection process does not change the results in most cases. The classification accuracy under the different pixel-selection proportions is shown in Figure 3.

Figure 3 shows the classification accuracy using SVM based on the bands selected by the ISMLP method under different pixel-selection proportions. Under the different pixel-selection proportions, the classification accuracy shown is the average value based on the 10 bands chosen by the ISMLP method. Because the bands selected using the pixel proportions from all pixels to 1/100 pixels are the same, the classification accuracy from all to 1/100 is stable. This shows that K-means can select more representative pixels by pixel clustering and can obtain almost the same accuracy as can be achieved using all pixels. However, using fewer pixels greatly reduces the amount of calculation required; therefore, K-means is an effective way to select pixels. However, as the proportion of selected pixels decreases further, the classification accuracy falls continuously because selecting too few pixels insufficiently represents the distribution and characteristics of pixels of different categories, and ultimately reduces the overall classification accuracy. Based on these results, performing a 1/100 pixel-selection with K-means achieves better classification accuracy and better efficiency than smaller pixel-selected proportions; thus, we adopted this pixel-selection proportion for the band selection experiments.

#### 3.1.4. Accuracy Evaluation of the Sea Ice Detection Method

**Figure 4:**
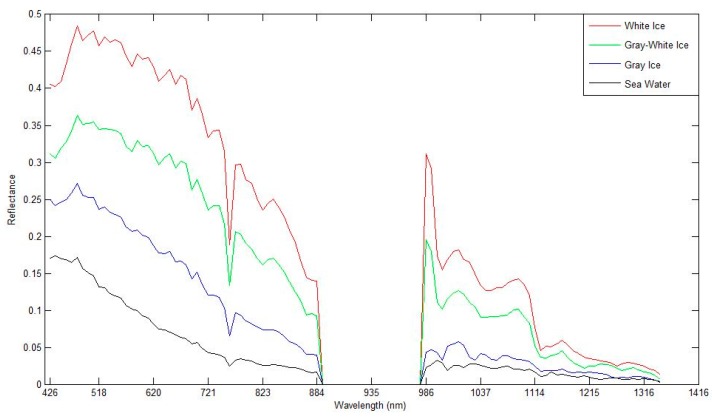
Spectral reflectance curves of sea ice types and sea water from hyperspectral data.

**Figure 5:**
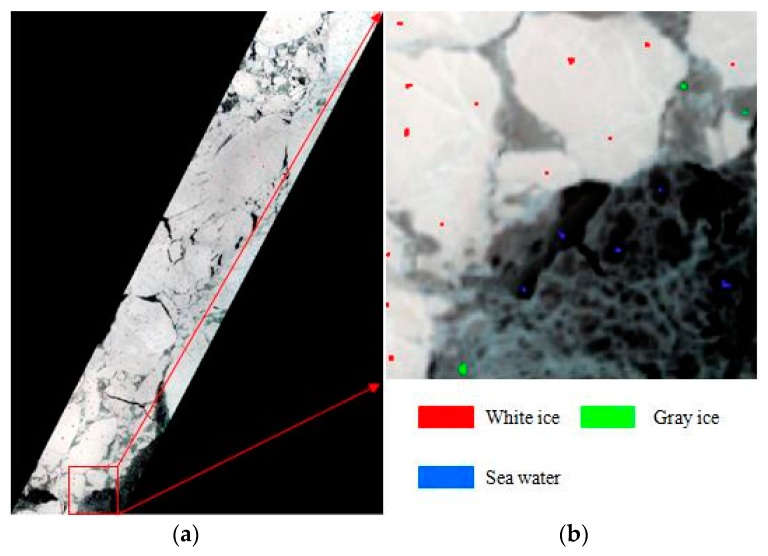
(**a**) Hyperspectral image marked with training samples (a true-color image composed of R: 29, G: 23, and B: 16); (**b**) A partial-enlargement image taken from (**a**).

Figure 4 provides the spectral reflectance curves for different ice types from hyperspectral images. We can see that the different types of sea ice can be discriminated by their spectral reflectance. According to the spectral reflectance curves of the hyperspectral data, three different categories are available: white ice, gray ice, and sea water. In this experiment, the image fusion of the original hyperspectral data was conducted based on Landsat data at first, and the spatial resolution of hyperspectral data was increased to 15 m. The available training samples (3303 samples) were collected in hyperspectral image interpretation, and then applied to classification based on SVM model. Example training samples are depicted in Figure 5a, Figure 5b is a partial enlargement image taken from Figure 5a. Because the quantity of test samples was far greater than the number of training samples, the entire image (except the background) can be taken as the test sample set. The classes and the corresponding sample numbers used in the experiments are shown in Table 2. 

**Figure 6:**
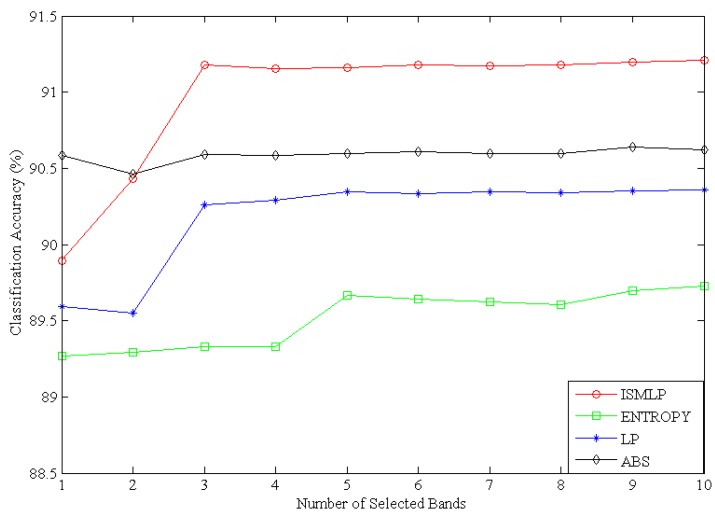
Classification accuracy curves of ISMLP, entropy, LP and ABS methods using the SVM classification model.

In this work, we used an SVM classifier with a radial basis function (RBF) kernel as a benchmark classifier to classify sea ice, and used the OAO method to solve the multiclass classification problem. The values for the regularization parameter C and the spread γ of the RBF kernel parameters are acquired by the cross-validation grid search method on the basis of the validation set. Finally, the best value of the parameter C is 32, the optimal kernel width parameter γ is found equal to 16. The accuracy of the classification model is assessed by using a confusion matrix. We chose the bands by the proposed ISMLP method, entropy, LP and ABS, then classified sea ice using the selected bands (from one band to ten bands) based on the SVM model, and assessed the accuracy of the classification by a confusion matrix. Here we took Landsat-8 data at the same time and same location as the validation data set. Aiming for the different number of selected bands, the classification accuracy of every method is the average value after five runs. Figure 6 shows a classification accuracy comparison of the ISMLP, entropy, LP and ABS methods using the SVM classification model.

As Figure 6 shows, the classification accuracy of ABS is higher than that of ISMLP in the first two bands; however, from a three-band selection to a ten-band selection, the classification accuracy of ISMLP is higher than that of ABS. This is because the ABS method selects the bands according to the band index in large-to-small order. It primarily considers the band information without considering either the similarity between the bands or the spectral continuity. Therefore, the selected bands are relatively concentrated and cannot cover the entire spectral range. Consequently, while ABS achieves a higher classification accuracy with the original two bands, its accuracy shows little change as subsequent bands are added. The LP method also depends on the original bands, but its original band selection is random. Thus, the classification accuracy of the traditional LP method is lower than that of the ISMLP method because of the influence of the original bands. The band selection method based on entropy takes only the amount of information in the bands into consideration; therefore, its classification results are also lower than the ISMLP method results. The ISMLP method not only considers the band information but also considers the similarity between the bands; therefore, its classification accuracy is higher than that of ABS in the subsequent (more than two) bands and is always higher than the traditional entropy and LP methods. Overall, the ISMLP method exhibits higher classification accuracy, indicating that the ISMLP method proposed in this paper performs better for classifying sea ice than do the traditional entropy, LP and ABS methods.

**Figure 7:**
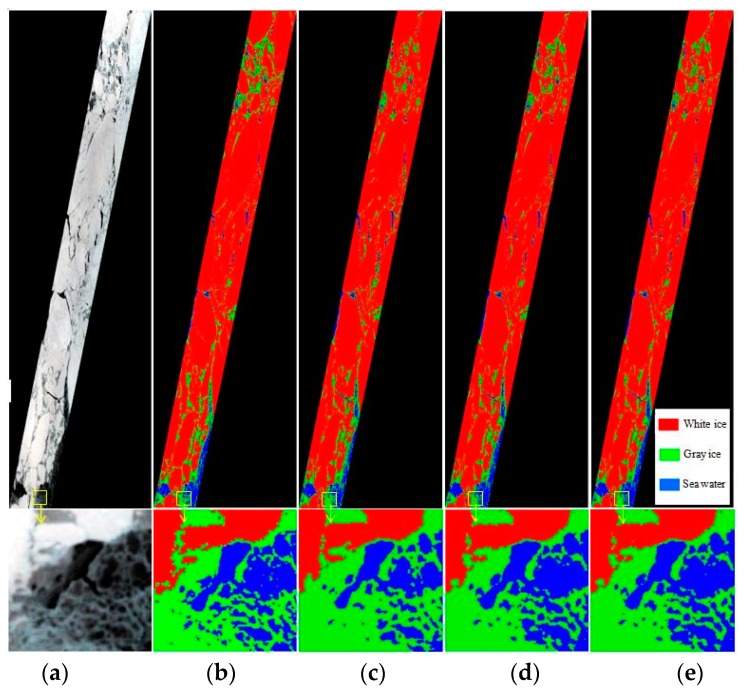
The results of the classification of band selection based on ISMLP method: (**a**) true color composite image, (**b**) classification results of Landsat-8, (**c**) classification results using two bands selected with the proposed ISMLP method, (**d**) classification results using three bands selected with the proposed ISMLP method, (**e**) classification results using four bands selected with the proposed ISMLP method.

Using the virtual dimension estimation method, the NSP yields the largest estimates in this experiment. When a given false alarm probability P_f_ occurs, the NSP method obtains VD estimates; the values of VD are all 3. It can be a reference for selected band numbers in the following analysis. From Figure 6, when the number of bands selected by the ISMLP method is greater than 3, the classification accuracy not only did not increase distinctly; it descended slightly. This illustrates that the VD provides a reasonable reference value for the optimal number of selected bands. Figure 7 shows the sea ice classification results based on the ISMLP method.

The results discussed above show that the performance of the ISMLP method is optimal because it takes into account both the amount of information the band contains and the cross-correlations between bands. To demonstrate the results more clearly, we also provide the partially enlarged details of the yellow boxed area shown in Figure 7. Figure 7a is a true color composite image, Figure 7b is the supervised classification results of Landsat-8, and Figure 7c–e shows the classification results when selecting 2, 3 and 4 bands, respectively, based on the ISMLP method. The overall classification accuracies obtained are 90.43, 91.18, and 91.16, respectively, and the kappa coefficients are 0.68, 0.71, and 0.7, respectively. These results indicate that the ISMLP method can choose a relatively better band subset than the other methods. The average classification accuracy is the highest when choosing 3 bands based on the ISMLP method, and the data dimensions are reduced from 92 to 3, which greatly reduces the calculation cost.

### 3.2. Experiment in Bohai Bay

#### 3.2.1. Data Description

**Figure 8:**
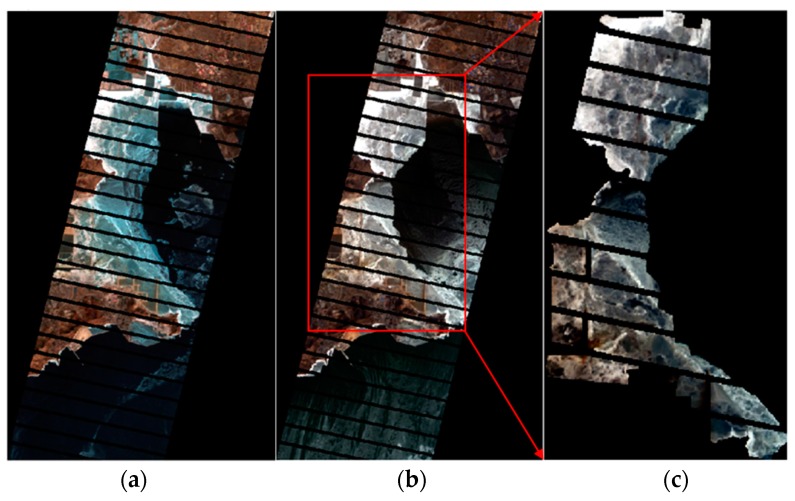
(**a**) Part of the original Landsat-7 data, (**b**) part of original hyperspectral data with mask processing, and (**c**) the experiment data (after removal of the land).

The data for the second experiment was acquired without cloud coverage from the EO-1 hyperspectral sea ice data of Bohai Bay on 23 January 2008. The upper left-hand corner of this area is 120°45′12′′ E, 41°39′7′′ N, and its bottom right-hand corner is 121°13′9′′ E, 39°44′42′′ N. The validation data consisted of Landsat-7 (with a spatial resolution of 15 m) sea ice data from the same location acquired on 26 January 2008. Because the scan line corrector (SLC) in the enhanced thematic mapper plus (ETM+) instrument of Landsat-7 failed on 31 May 2003, approximately 22% of the data in the Landsat-7 scene were missing [28]. Therefore, some strips in the Landsat-7 image, part of the original data, are missing data, as shown in Figure 8b. To effectively evaluate the data detection results, we used mask processing on the hyperspectral data to make it consistent with the Landsat-7 data. Part of the original hyperspectral data is shown in Figure 8a.

Because the acquisition time of the hyperspectral data shown in Figure 8a,b was three days apart from the Landsat-7 data, the peripheral glacial ice differs slightly due to the ocean current effects, but the coastal sea ice distribution is comparatively consistent. Therefore, we chose two pieces of data from the sea ice in the overlapping range as the experimental data (removal of land), as shown as in Figure 8c. The overall accuracy of the overlapping range is 95.4%.

#### 3.2.2. Preprocessing of Hyperspectral Data

According to the spectral characteristics of the hyperspectral sea ice data described in Section 3.1.2, 92 bands (from 8 to 57 and from 79 to 120) of hyperspectral data were as the band sources of the band selection in this experiment.

#### 3.2.3. Pixel Selection and Band Selection Based on the ISMLP Method

In this experiment, the higher spatial resolution Landsat-7 data from the same location as the EO-1 data were taken as the base band, and a comparative analysis of band selection was made using different pixel-selection proportions based on the K-means method. The band selection results are shown in Table 3.

**Figure 9:**
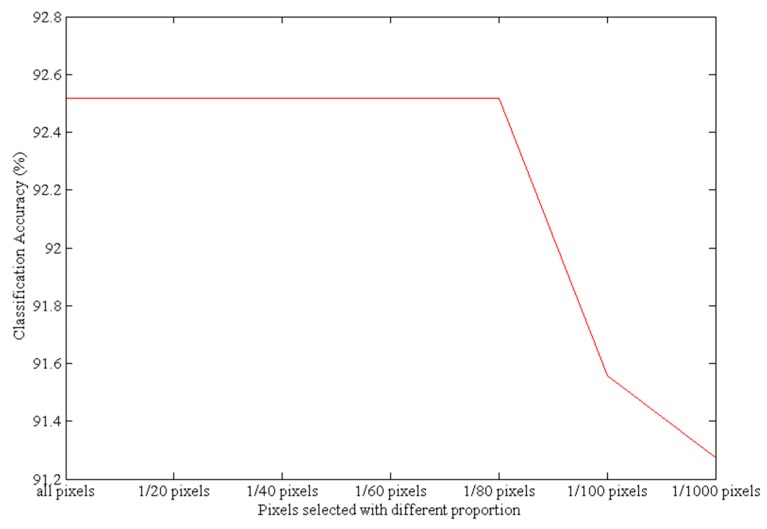
The classification accuracies resulting from different pixel-selection proportions.

As shown in Table 3, the selected bands are the same when using any of the pixel-selection proportions from all pixels to 1/80 for the K-means method, and the number of pixels can be reduced to 1/80 with no loss in accuracy. When using 1/100 pixels, although the band numbers change only slightly, the result is still unstable and greatly influences the classification accuracy when choosing subsequent bands. When using only 1/1000 pixels, the original band and the subsequent selected bands changed significantly. Consequently, selecting only 1/1000 pixels with the K-means method causes too few pixels to be selected and will decrease the performance of the band selection method because the reduced number of pixels cannot adequately describe the spatial characteristics of the bands. Therefore, we do not discuss this issue further in this paper. The classification accuracies obtained under the different pixel-selection proportions are shown in Figure 9.

Figure 9 shows the classification accuracies based on the band selection by the ISMLP method under different pixel-selection proportions with the SVM classification model. The classification values shown are the average values based on 10 bands chosen by the ISMLP method. The classification accuracy value is stable at proportions raging from all to 1/80; therefore, we chose 1/80 as the pixel-selection proportion in this experiment.

#### 3.2.4. Accuracy Evaluation of Sea Ice Detection

**Figure 10:**
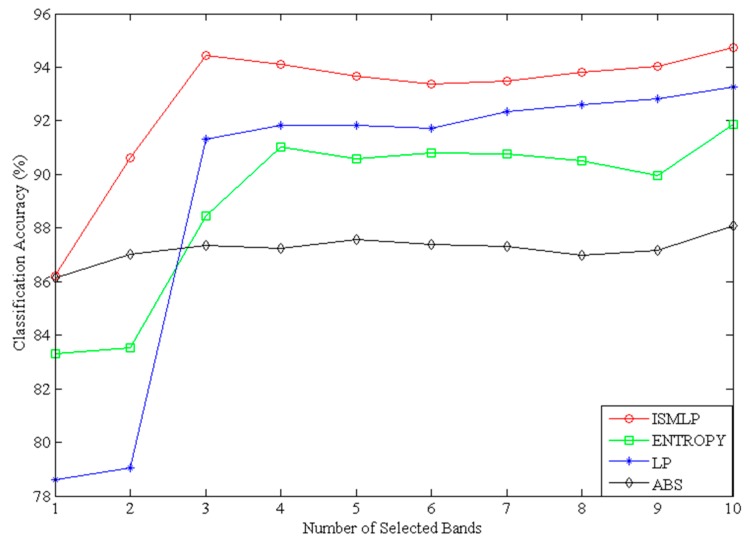
Classification accuracy curves of the ISMLP, Entropy, LP and ABS methods using the SVM classification model.

Based on the spectral reflectance curves of the hyperspectral data, three different categories are available in the second experiment, namely, white ice, gray-white ice, and gray ice. Similar to the previous experiment, we chose the bands by the ISMLP, entropy, LP and ABS methods, classified sea ice based on SVM model, and we also used a confusion matrix to evaluate the sea ice detection accuracy based on the reference image (Landsat-7 data) in this experiment. The classification accuracy of every method is the average value after five runs also. Figure 10 shows a comparison of the classification accuracy achieved by the ISMLP, entropy, LP and ABS methods using the SVM classification model.

As shown in Figure 10, we obtained results similar to those from the Baffin Bay experiment; except that, here, the classification accuracy of ISMLP is always higher than the ABS, entropy and LP methods. Overall, the ISMLP method exhibits better classification accuracy, which shows that the ISMLP method proposed in this paper performs better at detecting sea ice than the traditional entropy, LP and ABS methods. 

On the other hand, when different false alarm probabilities are given, the values of VD are all 3, and when the band number selected by the ISMLP method is larger than 3, the classification accuracy drops distinctly. This further illustrates that the VD is a reasonable reference value when selecting the number of bands.

**Figure 11:**
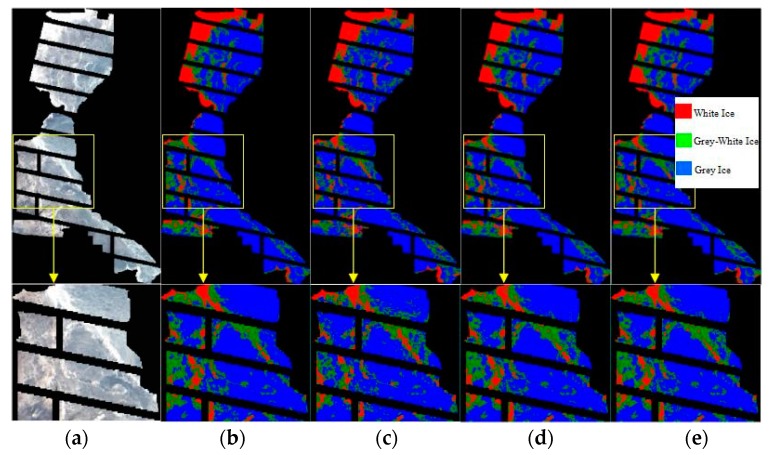
The classification resulting from band selection based on the ISMLP method: (**a**) true color composite image, (**b**) classification results of Landsat-7, (**c**) classification results using two bands selected with the proposed ISMLP method, (**d**) classification results using three bands selected with the proposed ISMLP method, and (**e**) classification results using four bands selected with the proposed ISMLP method.

Figure 11 shows the classification results from band selection based on the ISMLP method. The figure structure is consistent with the results of first experiment (Figure 7). The overall accuracy values of the SVM classification, determined by verification against the Landsat-7 data, are 89.67, 94.22, and 93.95, and the Kappa coefficients are 0.85, 0.90, and 0.89, respectively. These results also indicate that the ISMLP method can achieve good performance when classifying sea ice from hyperspectral images.

## 4. Conclusions

The goal of this work was to improve the performance of hyperspectral sea ice detection. In this paper, we proposed an improved similarity measurement method for dimensionality reduction of sea ice hyperspectral images to obtain the optimal band combination, which contains both a large amount of information and exhibits low similarity. Our proposed method is compared with the LP, entropy and ABS methods with regard to classification accuracy in two experiments. According to the experimental results, overall, the ISMLP method performs better in detecting sea ice than the traditional methods. From analyzing the experiment results, we can summarize as follows:

- Considering each band’s information and the similarity between bands, the proposed ISMLP method produces the best classification accuracy compared to the traditional methods while also greatly reducing the data dimensions.
- Considering the spectral characteristics of sea ice, this work chose bands that have good spectral characteristics and separability and applied the band selection of the hyperspectral image to only those bands. This approach effectively reduces the scope of the original bands and enhances the efficiency of the method.
- Considering the high spatial correlations in the hyperspectral images, we selected pixels based on K-means clustering and analyzed the changes in the classification accuracy resulting from pixel selection at different proportions. The results revealed that selecting a proportion of approximately 1/100 pixels with K-means maintains the balance between efficiency and performance. That is, the 1/100 pixel-selection proportion reduced the computational cost while simultaneously achieving higher classification accuracy than other pixel-selection proportions.

According to the results of the experiments, our method obtains superior performance with fewer dimensions and higher efficiency. It should be noted that the influence of snow coverage was not considered in this paper; in future studies, we plan to combine our method with an image texture analysis method to eliminate the influence of weather conditions.

## Figures and Tables

**Table 1:** Bands selected by the ISMLP method under the different pixel-selection proportion.

Pixel Select Proportions	Bands Selected by ISMLP Method
All pixels	16, 118, 84, 42, 79, 40, 82, 8, 89, 85
1/20	16, 118, 84, 42, 79, 40, 82, 8, 89, 85
1/40	16, 118, 84, 42, 79, 40, 82, 8, 89, 85
1/60	16, 118, 84, 42, 79, 40, 82, 8, 89, 85
1/80	16, 118, 84, 42, 79, 40, 82, 8, 89, 85
1/100	16, 118, 84, 42, 79, 40, 82, 8, 89, 85
1/1000	16, 120, 84, 79, 42, 40, 82, 8, 90, 85
1/10,000	19, 120, 84, 79, 42, 40, 81, 8, 57, 91

**Table 2:** Number of training samples in each class.

Class	Training Samples
White ice	2031
Gray ice	723
Sea water	549
Total	3303

**Table 3:** Bands selected by the ISMLP method under different pixel-selection proportion.

Pixel Select Proportions	Bands Selected by ISMLP Method
All pixels	21, 120, 83, 8, 79, 81, 42, 85, 94, 9
1/20	21, 120, 83, 8, 79, 81, 42, 85, 94, 9
1/40	21, 120, 83, 8, 79, 81, 42, 85, 94, 9
1/60	21, 120, 83, 8, 79, 81, 42, 85, 94, 9
1/80	21, 120, 83, 8, 79, 81, 42, 85, 94, 9
1/100	21, 120, 83, 8, 79, 81, 42, 94, 86, 9
1/1000	20, 120, 84, 79, 8, 82, 39, 91, 9, 94
